# A Nck‐associated protein 1‐like protein affects drought sensitivity by its involvement in leaf epidermal development and stomatal closure in rice

**DOI:** 10.1111/tpj.14288

**Published:** 2019-03-18

**Authors:** Lichao Huang, Long Chen, Lan Wang, Yaolong Yang, Yuchun Rao, Deyong Ren, Liping Dai, Yihong Gao, Weiwei Zou, Xueli Lu, Guangheng Zhang, Li Zhu, Jiang Hu, Guang Chen, Lan Shen, Guojun Dong, Zhenyu Gao, Longbiao Guo, Qian Qian, Dali Zeng

**Affiliations:** ^1^ State Key Laboratory of Rice Biology China National Rice Research Institute Hangzhou 310006 China; ^2^ College of Chemistry and Life Sciences Zhejiang Normal University Jinhua 321004 China

**Keywords:** drought sensitivity, stomatal closure, abscisic acid, stomatal density, cuticle, rice (*Oryza sativa* L.)

## Abstract

Water deficit is a major environmental threat affecting crop yields worldwide. In this study, a drought stress‐sensitive mutant *drought sensitive 8* (*ds8*) was identified in rice (*Oryza sativa* L.). The *DS8* gene was cloned using a map‐based approach. Further analysis revealed that *DS8* encoded a Nck‐associated protein 1 (NAP1)‐like protein, a component of the SCAR/WAVE complex, which played a vital role in actin filament nucleation activity. The mutant exhibited changes in leaf cuticle development. Functional analysis revealed that the mutation of *DS8* increased stomatal density and impaired stomatal closure activity. The distorted actin filaments in the mutant led to a defect in abscisic acid (ABA)‐mediated stomatal closure and increased ABA accumulation. All these resulted in excessive water loss in *ds8* leaves. Notably, antisense transgenic lines also exhibited increased drought sensitivity, along with impaired stomatal closure and elevated ABA levels. These findings suggest that *DS8* affects drought sensitivity by influencing actin filament activity.

## Significance Statement

*DS8* encodes a NAP1‐like protein that affects drought sensitivity by its involvement in leaf epidermal development and stomatal closure in rice, revealing a molecular mechanism concerning the SCAR/WAVE‐Arp2/3 complex in the drought stress response.

## Introduction

Adverse climatic conditions, such as drought, threaten rice growth and production. To adapt to drought stress, plants have evolved various mechanisms involving adaptive changes at the morphological, physiological and molecular levels. Characteristics of the leaf epidermis are important indicators of plant drought tolerance (Fang and Xiong, 2015).

In rice, the epidermal cell wall is covered by a hydrophobic cuticle on the outside and embedded with silica beneath the cuticle (Kunst and Samuels, 2003; Ma and Yamaji, 2008). The cuticle, composed of cutin and wax, is an indispensable barrier that prevents non‐stomatal water loss (Mao *et al*., 2012). Mutants with changes in cuticle wax components, such as *wsl1*,* wsl3*,* wsl4*,* cfl1* and *dwa1*, have increased sensitivity to drought stress (Yu *et al*., 2008; Wu *et al*., 2011; Zhu and Xiong, 2013; Gan *et al*., 2016; Wang *et al*., 2017). The silica‐embedded cuticular papillae (CP) also help reduce cuticular transpiration in rice (Yoshida *et al*., 1962; Agarie *et al*., 1998; Epstein, 1999; Gao *et al*., 2005).

Stomata, which play a role in water and gas exchange, are responsible for approximately 90% of the water loss that occurs in the leaf epidermis (Buckley, 2005). Stomatal density and stomatal aperture are therefore important indices used to evaluate drought resistance in plants. Overexpression lines of *OsDT11* and *SDD1* display dramatically enhanced drought tolerance due to their reduced stomatal densities (Yoo *et al*., 2010; Li *et al*., 2016). Stomata generally close in response to low environmental humidity to prevent excessive water loss (Pantin and Blatt, 2018). Genes that positively modulate stomatal closure can improve the survival rates of plants during drought stress (Huang *et al*., 2009; Jin *et al*., 2013; Dey *et al*., 2016; Gao *et al*., 2018).

Abscisic acid (ABA) induces stomatal closure through a complex signaling network (Munemasa *et al*., 2015; Matsuda *et al*., 2016; Sussmilch and McAdam, 2017). The perception of the ABA signal by ABA receptors activates downstream plasma membrane Ca^2+^ channels and K^+^ efflux channels, resulting in stomatal closure (Munemasa *et al*., 2015). A decrease in the ABA content in guard cells dampens the response of leaf stomata to ABA stimulation, leading to reduced stomatal closure, followed by increased sensitivity to water loss (Kang *et al*., 2010; Matsuda *et al*., 2016). ABA also regulates stomatal aperture by driving reactive oxygen species (ROS) biosynthesis (Mittler and Blumwald, 2015; Sierla *et al*., 2016). Rice plants with elevated H_2_O_2_ levels in guard cells exhibit reduced stomatal aperture and increased tolerance to water deficit (Liu *et al*., 2016; Hu *et al*., 2017).

Most studies on ABA‐mediated stomatal closure have focused on the functions of ABA receptors or ion channels in guard cells (Park *et al*., 2009; Geiger *et al*., 2010; Guo *et al*., 2011; Zhao *et al*., 2016; Müller *et al*., 2017). Some studies have pointed to a relationship between the ABA‐signaling network and actin filaments (Li *et al*., 2014). Actin filaments are major components of the complex cytoskeleton, and participate in various cellular events affecting plant growth and development (McCurdy *et al*., 2001; Thomas and Staiger, 2014). During stomatal closure, the actin filaments in guard cells undergo a depolymerization‐reintegration process that functions upstream of stomatal movement (Eun and Lee, 2000; Gao *et al*., 2009). To ensure successful stomatal closure, the radially oriented actin filaments in open stomata must depolymerize, become randomly distributed, and reintegrate in longitudinal actin cables (Dong *et al*., 2001; Lemichez *et al*., 2001; Zhang *et al*., 2007; Higaki *et al*., 2010; Zhao *et al*., 2011). Suppressed actin filaments dynamics in *Arabidopsis thaliana* guard cell delays ABA‐mediated stomatal closure and results in hypersensitivity to drought stress (Zhao *et al*., 2011). The Arp2/3 complex, comprising seven elements (ARP2, ARP3, ARPC1, ARPC2, ARPC3, ARPC4 and ARPC5), is activated by the SCAR/WAVE complex to increase the efficiency of actin filaments nucleation and to initiate new actin filaments branching (Pollard, 2007; Yanagisawa *et al*., 2015). The SCAR/WAVE complex consists of five evolutionarily conserved subunits, namely ABI, BRICK1/HSPC300, NAP1/NAP125, PIR/SRA1 and SCAR (Le *et al*., 2006; Mendoza, 2013; Zhou *et al*., 2016). The SCAR/WAVE complex members can enhance the Arp2/3 complex‐mediated nucleation efficiency of actin filaments *in vitro* and *in vivo* (Welch and Mullins, 2002; Bai *et al*., 2015). All those results indicated that the SCAR/WAVE‐ Arp2/3 complex may be involved in ABA‐mediated stomatal closure.

Enormous effort has been devoted to elucidating the role of the SCAR/WAVE‐Arp2/3 complex in the formation and development of leaf epidermal cells by involvement in actin filaments modeling over the past decades (Frank and Smith, 2002; Djakovic *et al*., 2006; Facette *et al*., 2015); however, few studies have focused on the function of SCAR/WAVE complex in plant responses to environmental stress, especially drought stress. In our study, the Nck‐associated protein 1 (NAP1)‐like protein coding gene *DROUGHT SENSITIVE 8* (*DS8*) was hypothesized to affect drought sensitivity by influencing actin filament activity. The study was designed to find out how *DS8* affects drought sensitivity in rice plant, focusing on the impacts of *DS8* on indicators related to drought sensitivity, including cuticle and stoma. Cytochalasin B (CB) and sodium tungstate treatments were performed to preliminarily analyze the relationship between ABA and actin filaments activity during ABA‐induced stomatal closure.

## Results

### *ds8* is sensitive to drought stress

**Figure 1:**
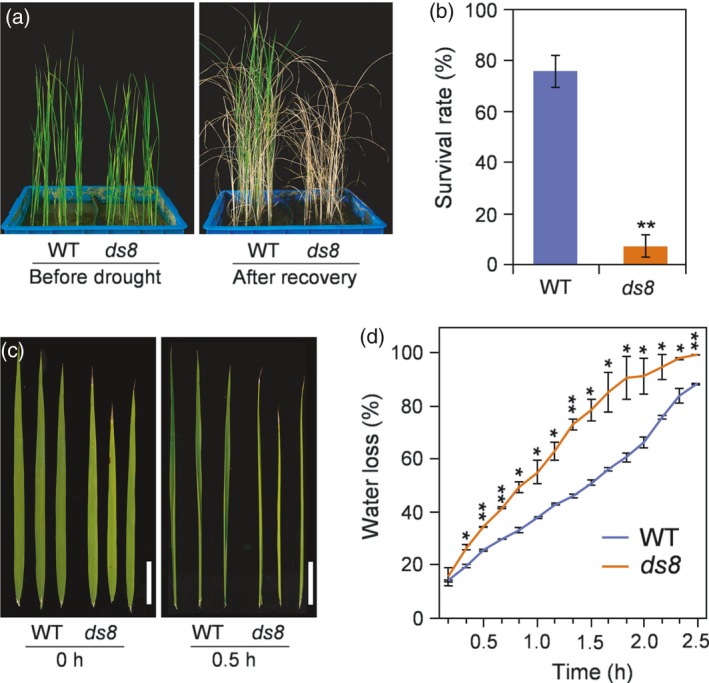
The *ds8* mutant shows increased sensitivity to drought stress. (a) Wild‐type (WT) and *ds8* seedlings subjected to drought stress. (b) Survival rates of WT versus *ds8* after recovery from drought stress. Data are represented as mean ± SD (*n *= 3). **P ≤ 0.01; Student's *t*‐test. (c) Phenotypes of WT and *ds8* leaves at 0 and 30 min after excision at the heading stage. Scale bar: 5 cm. (d) Water loss rate of WT and *ds8* leaves excised from plants at the heading stage. Comparison was made between WT and *ds8* at each time point. Data are represented as mean ± SD (*n *= 3). **P ≤ 0.01; *P ≤ 0.05; Student's *t*‐test.

The *ds8* mutant was screened under drought stress conditions from a Nipponbare (NPB, *japonica*) rice mutant pool generated by ethyl methanesulfonate mutagenesis. A drought stress test indicated that *ds8* seedlings were more sensitive to drought stress than the wild‐type (Figure 1a). After recovery from drought treatment, the survival rate of *ds8* was only 9.4% that of wild‐type (Figure 1b). In addition, when excised flag leaves were placed in an incubator at 60% humidity, *ds8* leaves curled quickly (Figure 1c) and showed a higher water loss rate than wild‐type leaves (Figure 1d). We also investigated major agronomic traits related to grain yield in dry environmental conditions. Under drought conditions, *ds8* suffered higher decline rates in agronomic traits such as tiller number, grain number per panicle, filled grains per panicle and grain yield per plant than the wild‐type (Figure S1; Table S1).

### Map‐based cloning of *DS8*

Reciprocal crosses between *ds8* and three varieties (9311, ZF802 and CJ06) of wild‐type rice plants were carried out. Plants from all six F_1_ populations exhibited normal phenotypes. In all F_2_ populations, the ratio of wild‐type to *ds8* mutant phenotypes in every combination was ~3:1 (Table S2).

**Figure 2:**
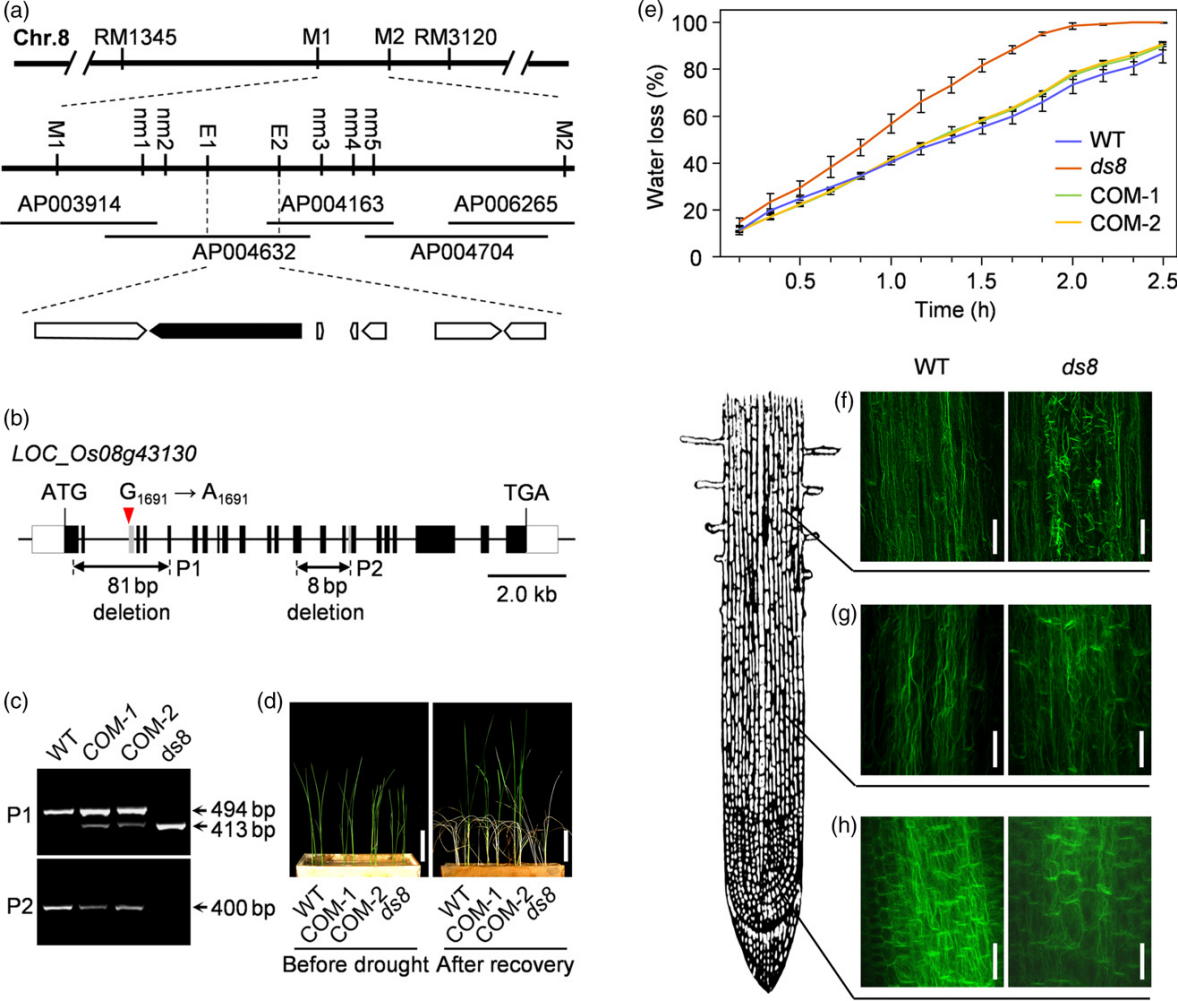
Map‐based cloning of *DS8*. (a) Fine mapping of *ds8*. The associated markers and BACs are labeled. (b) *DS8* gene structure. Solid rectangles and lines indicate exons and introns, respectively. Gray rectangle represents the missing region of the exon. The mutation site and primers used to detect the missing regions are also labeled. (c) Identification of transgenic complementation lines using the markers labeled in (b). (d) Transgenic complementation seedlings subjected to drought stress. Scale bar: 5 cm. (e) Water loss rates of excised leaves harvested from transgenic complementation lines at the heading stage. Data are represented as mean ± SD (*n *= 3). The least significance difference test was applied at 0.05 probability level (Table S4). (f–h) Observation of actin filaments in (f) the maturation zone, (g) the elongation zone and (h) the meristematic zone of wild‐type (WT; left) and *ds8* (right) root tips. Scale bar: 20 μm.

We performed map‐based cloning, and mapped *DS8* to chromosome 8 between markers RM1345 and RM3120 (Figure 2a). The primer sequences used are listed (Table S3). Furthermore, using 2319 homozygous mutant plants, *DS8* was narrowed down to a 53.7‐kb genomic region between markers E1 and E2. There were seven predicted open reading frames (ORFs) in this region, according to the Rice Genome Annotation Project (RGAP). We sequenced these predicted ORFs and identified a single‐base substitution (G to A) at the last nucleotide of the second intron in *LOC_Os08g43130* in *ds8*. This mutation caused a premature stop codon due to the deletion of the entire third exon (81 bp) and part of the 17th exon (8 bp; Figure 2b).

A genetic complementation test was arranged to *ds8* mutant and 22 positive *proDS8:DS8* transformants were obtained, all transformants exhibited wild‐type levels of drought tolerance, confirming the identity of *DS8* as *LOC_Os08g43130* (Figures 2c,d and S2). Moreover, the leaf water loss rate of the transgenic plants was similar to that of the wild‐type (Figure 2e; Table S4).

We carried out quantitative reverse‐transcription polymerase chain reaction analysis of *DS8* transcript levels in various tissues at the booting stage. *DS8* was widely expressed in panicle, culm, sheath, root and leaf tissue (Figure S3a). We also detected GUS (β‐glucuronidase) activity in these tissues in *proDS8:GUS* transgenic rice plants (Figure S3b–f). Analysis of the subcellular localization of DS8‐eGFP in rice protoplasts and *pro35S:DS8‐eGFP* transgenic plants indicated that DS8 was localized to the cytoplasm (Figure S3g,h).

### *DS8* encodes a NAP1‐like protein involved in actin filaments function

Sequence analysis showed that *DS8* comprised 23 exons and 22 introns, and encoded a 1359 amino‐acid protein (Figure 2b). A protein–protein BLAST search against the NCBI database using the deduced DS8 protein sequence as a query revealed that it was a NAP1‐like protein. We selected 25 DS8 homologs from various species to determine the evolutionary relationship between DS8 and these proteins, finding that the DS8 homologs were conserved in both monocots and dicots (Figure S4). Protein sequence alignment revealed that DS8 shares 86.28% amino acid identity with homologs from *Hordeum vulgare*,* Sorghum bicolor*,* Zea mays* and *A. thaliana* (Figure S5).

The organization of actin filaments in the seedling roots of *ds8* and wild‐type plants was observed. The actin filaments were complete and regularly arranged in wild‐type. In *ds8*, disordered actin filaments were observed at the meristematic zone, became more severe during cell growth from the meristematic zone to maturation zone, and appeared incomplete and cluttered in the maturation zone (Figure 2f–h).

### *DS8* is involved in leaf cuticle development

**Figure 3:**
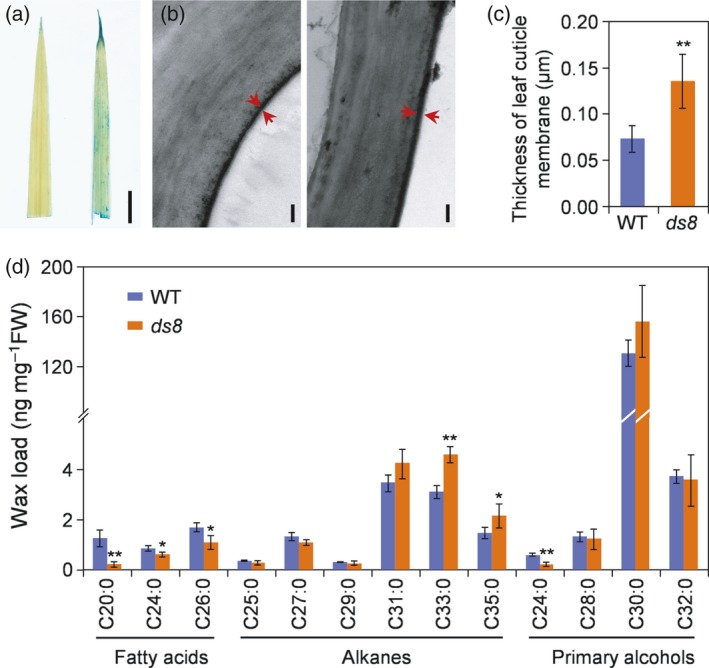
*DS8* is involved in leaf cuticle development. (a) Toluidine blue staining of wild‐type (WT; left) and *ds8* (right) leaves. Scale bar: 1 cm. (b) Transmission electron microscopy (TEM) analysis of the cuticular layer in WT (left) and *ds8* (right) leaf epidermis. Red arrows point to the cuticular layer. Scale bar: 0.2 μm. (c) Comparison of cuticular layer thickness between WT and *ds8*. Data are represented as mean ± SD (*n *= 6). **P ≤ 0.01; Student's *t*‐test. (d) Cuticle wax composition and loads in WT and *ds8*. Data are represented as mean ± SD (*n *= 4). **P ≤ 0.01; *P ≤ 0.05; Student's *t*‐test.

We stained the leaves of wild‐type and *ds8* plants with toluidine blue, which can roughly reflect the integrity of the cuticle on the leaf surface. Wild‐type leaves successfully resisted toluidine blue staining, whereas *ds8* leaves were partially stained by the blue dye (Figure 3a). Transmission electron microscopy (TEM) analysis showed that the cuticular layer of the leaf epidermis in *ds8* was significantly thicker (nearly 1.85‐fold) than that of wild‐type (Figure 3b,c). However, the less osmiophilic membrane of *ds8* exhibited reduced electron density, as revealed by its lighter coloring (Figure 3b). We also compared the wax components of the leaf cuticles of wild‐type and *ds8* plants. The levels of very‐long‐chain fatty acids, such as C20:0, C24:0 and C26:0, were reduced in *ds8*, whereas the levels of alkanes such as C33:0 and C35:0 were significantly higher in *ds8* compared with the wild‐type (Figure 3d).

The cell wall components in wild‐type and *ds8* leaves were also measured. Pectin, hemicellulose I and cellulose levels were significantly lower in *ds8* leaves than in wild‐type leaves (Figure S6a). The levels of monosaccharides such as arabinose, mannose, fructose, glucose and galactose were also significantly reduced in *ds8* leaves (Figure S6b).

### *DS8* affects stomatal density

**Figure 4:**
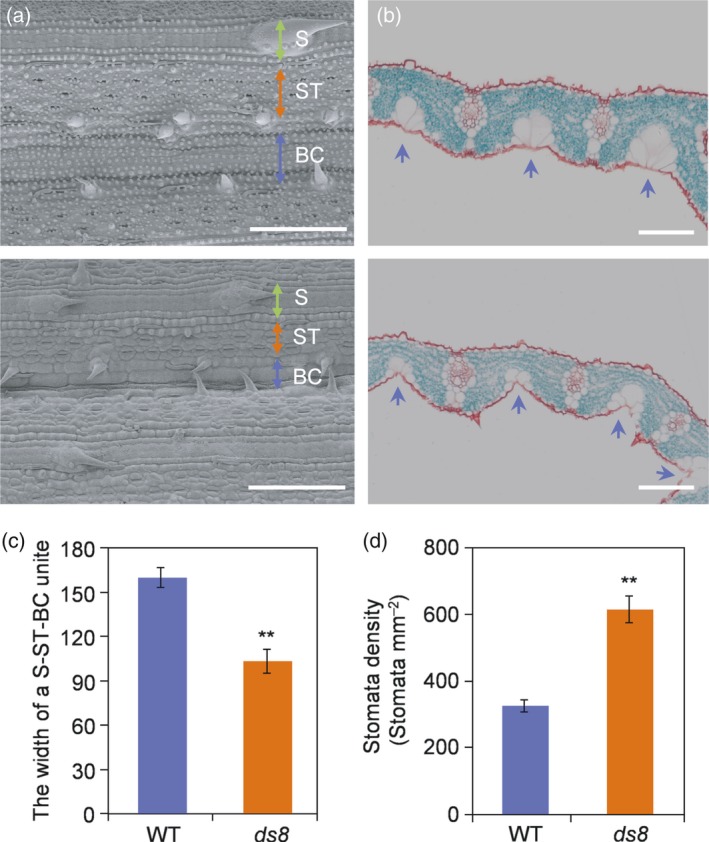
*DS8* affects stomatal density. (a) Scanning electron microscopy (SEM) analysis of upper leaf epidermis in wild‐type (WT; upper panel) and *ds8* (lower panel). S, silica‐phellem band; ST, stomatal band; BC, bulliform cell band. Scale bar: 100 μm. (b) Cross‐sections of WT (upper panel) and *ds8* (lower panel) leaves. Blue arrows point to grooves between two vascular bundles. Scale bar: 100 μm. (c) The width of a S‐ST‐BC unit in WT and *ds8* leaf epidermis. Data are represented as mean ± SD (*n *= 6). **P ≤ 0.01; Student's *t*‐test. (d) Comparison of stomatal density between WT and *ds8*. Data are represented as mean ± SD (*n *= 6). **P ≤ 0.01; Student's *t*‐test.

Observation of leaf ultrastructure revealed that the bulliform cell band on the upper leaf epidermis of *ds8* was more wrinkled than that of the wild‐type (Figure 4a). Cross‐sections of *ds8* leaves exhibited shrunken bulliform cells, leaving a groove between two vascular bundles (Figure 4b). The width of the S‐ST‐BC unit in the leaf epidermis of wild‐type and *ds8* was measured. The width of the S‐ST‐BC unit in *ds8* leaf was reduced to approximately 64.4% that of the wild‐type (Figure 4c). The stomatal density in *ds8* leaves showed an obvious increase compared with the wild‐type (Figure 4d).

### *DS8* affects stomatal closure and abscisic acid accumulation

**Figure 5:**
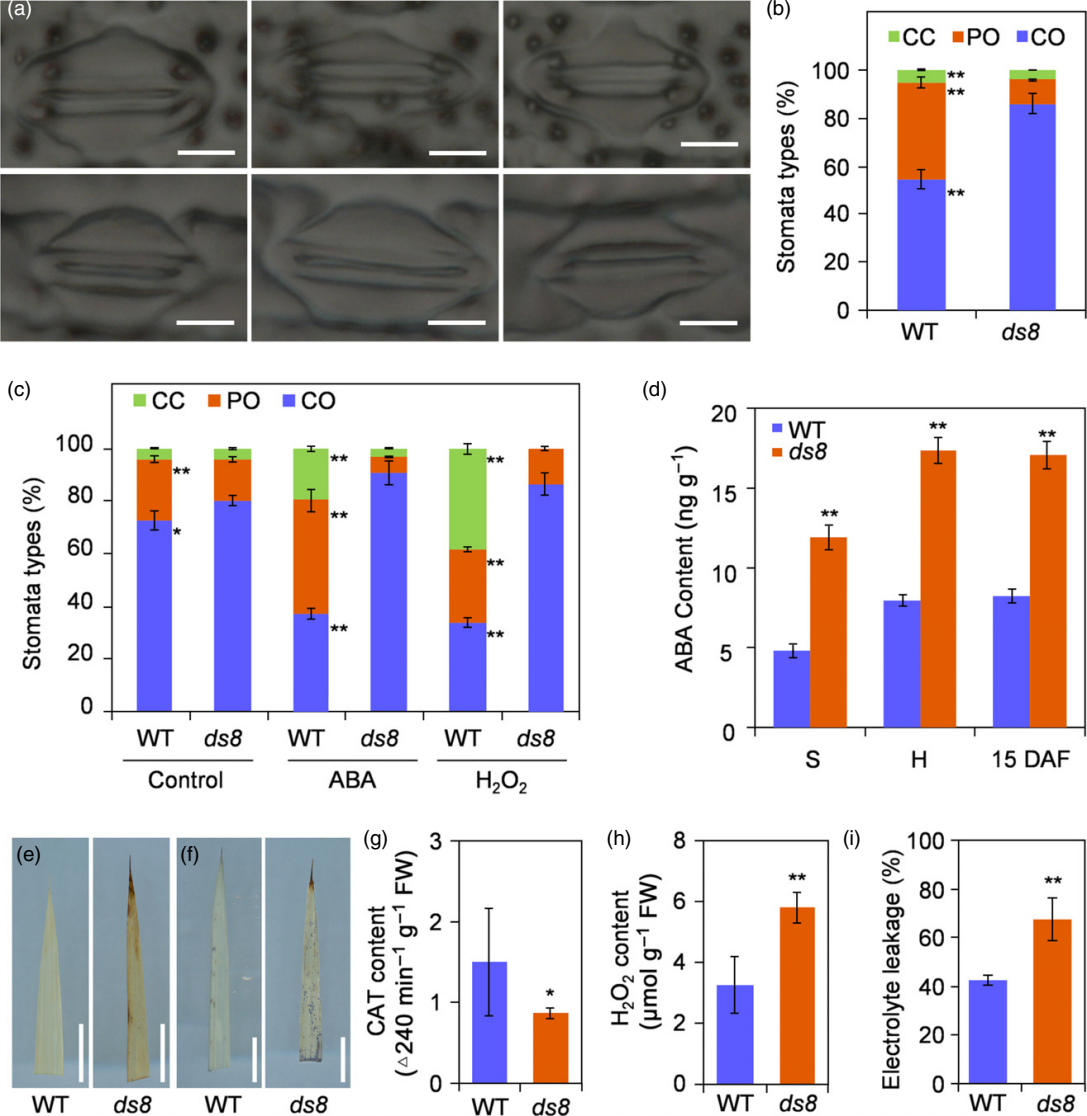
*DS8* affects stomatal closure and abscisic acid (ABA) accumulation. (a) Three different stomatal aperture types in wild‐type (WT; upper panels) and *ds8* (lower panels): completely open (CO; left), partially open (PO; middle) and completely closed (CC; right). Scale bar: 5 μm. (b) Percentage of each type of stoma in WT and *ds8*. At least 100 stomata from five individual plants were analyzed for WT and *ds8*, respectively. Comparison was made between WT and *ds8* of the same stoma type. Data are represented as mean ± SD. **P ≤ 0.01; Student's *t*‐test. (c) Percentage of each type of stoma in WT and *ds8* during ABA and H_2_O_2_ treatment. Leaves with maximum stomatal aperture status were used as the control. At least 100 stomata from five individual plants were analyzed per treatment. Comparison was made between WT and *ds8* of the same stoma type in each treatment. Data are represented as mean ± SD. **P ≤ 0.01; *P ≤ 0.05; Student's *t*‐test. (d) Foliar ABA content in WT and *ds8* during different growth phases in the paddy field. S, seedling stage; H, heading stage; 15 DAF, 15 days after flowering. Data are represented as mean ± SD (*n *= 3). **P ≤ 0.01; Student's *t*‐test. (e) 3,3’‐Diaminobenzidine (DAB) staining of excised leaves from WT (left) and *ds8* (right) plants. Scale bar: 1 cm. (f) Nitrotetrazolium chloride (NBT) staining of excised leaves from WT (left) and *ds8* (right) plants. Scale bar: 1 cm. (g) Catalase (CAT) activity in WT and *ds8* leaves. Data are represented as mean ± SD (*n *= 3). *P ≤ 0.01; Student's *t*‐test. (h) H_2_O_2_ content in WT and *ds8* leaves. Data are represented as mean ± SD (*n *= 3). **P ≤ 0.01; Student's *t*‐test. (i) Comparison of electrolyte leakage between WT and *ds8*. Data are represented as mean ± SD (*n *= 3). **P ≤ 0.01; Student's *t*‐test.

The stomatal aperture status on the upper leaf epidermis was examined. In general, the stomatal aperture status was categorized into three types (Figure 5a): completely open (CO); partially open (PO); and completely closed (CC). The proportion of CO stomata in seedling leaf was significantly higher in *ds8* than in the wild‐type (approximately 86 and 54%, respectively) under field conditions. Meanwhile, the proportions of PO and CC stomata were significantly lower in *ds8* than in the wild‐type (Figure 5b). We then treated wild‐type and *ds8* leaves with ABA and H_2_O_2_, which can promote stomatal closure. After being opened to a maximum level, the proportion of CO‐type stomata was significantly reduced in wild‐type leaves during ABA or H_2_O_2_ treatment, whereas the degree of CO‐type stomata in *ds8* leaves exhibited an unexpected increase (Figure 5c). In addition, the ABA levels in wild‐type and *ds8* leaves during various growth phases in the paddy field were measured. The endogenous ABA content was significantly higher in *ds8* than in the wild‐type. The ABA content remained high in *ds8* leaves throughout the growing season (Figure 5d). Furthermore, the *ds8* mutant exhibited withered leaf tips under natural field conditions (Figure S7a,b).

During the filling stage, elevated ROS contents were detected in *ds8* flag leaves, as revealed by 3,3ʹ‐diaminobenzidine (DAB) and nitrotetrazolium blue chloride (NBT) staining (Figure 5e,f). We also detected higher H_2_O_2_ production and significantly reduced catalase (CAT) activity in *ds8* flag leaves compared with wild‐type (Figure 5g,h). Significantly higher rates of electrolyte leakage were detected in *ds8* leaves than in wild‐type leaves (Figure 5i). TEM analysis showed that the number of dark osmiophilic globules and starch grains, which usually increased during leaf senescence, significantly increased in *ds8* mesophyll cells at the filling stage compared with the wild‐type (Figure S7c). The chlorophyll content and photosynthesis rate was significantly reduced in *ds8* leaves compared with the wild‐type (Figure S7d,e).

### *DS8*‐antisense transgenic lines exhibit increased drought sensitivity

**Figure 6:**
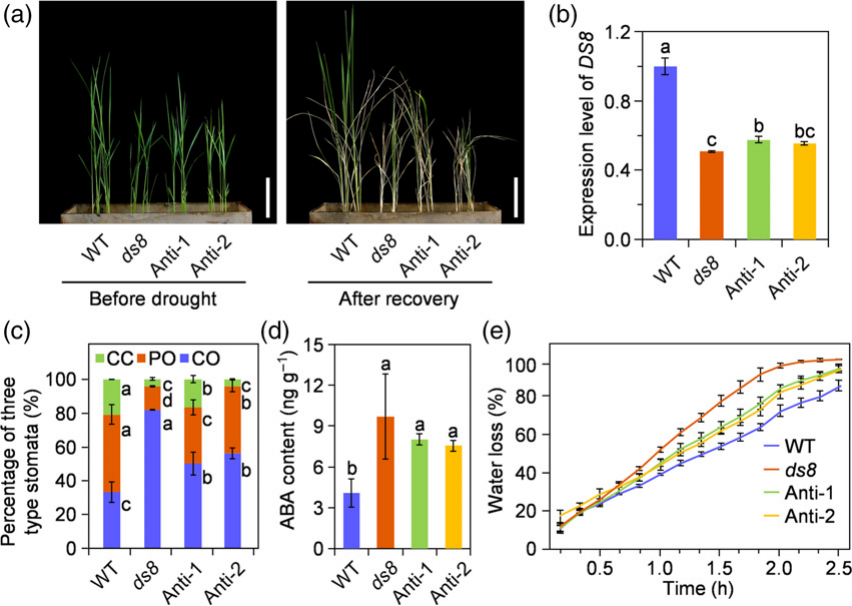
*DS8* antisense transgenic lines exhibit increased drought sensitivity. (a) *DS8* antisense transgenic lines subjected to drought stress. Scale bar: 5 cm. (b) *DS8* transcript level in wild‐type (WT),* ds8* and antisense transgenic lines. Data are represented as mean ± SD (*n *= 3). Different letters above columns indicate statistically significant differences (least significance difference test, *P *≤ 0.05). (c) Percentage of each type of stoma in WT,* ds8* and *DS8* antisense transgenic lines. CO, completely open; PO, partially open; CC, completely closed. Data are represented as mean ± SD. At least 100 stomata from five individual plants were analyzed, respectively, in WT,* ds8* and each antisense transgenic line. Different letters next to columns indicate statistically significant differences between WT,* ds8* and antisense transgenic lines of the same stoma type (least significance difference test, *P *≤ 0.05). (d) Abscisic acid (ABA) content in WT,* ds8* and antisense transgenic lines. Data are represented as mean ± SD (*n *= 3). Different letters above columns indicate statistically significant differences (least significance difference test, *P *≤ 0.05). (e) Water loss rates of excised leaves harvested from WT,* ds8* and antisense transgenic plants at the heading stage. Data are represented as mean ± SD (*n *= 3). The least significance difference test was applied at 0.05 probability level (Table S5).

The *DS8*‐antisense transgenic lines were generated. Two antisense transgenic lines with clearly reduced *DS8* transcript levels showed increased sensitivity to drought stress (Figure 6a,b). Compared with the wild‐type, the proportion of CO‐type stomata and ABA content were both higher in the *DS8*‐antisense transgenic seedlings under normal field conditions (Figure 6c,d). Excised leaves harvested from these lines showed an intermediate water loss rate between wild‐type and *ds8* levels (Figure 6e; Table S5). These results confirmed that *DS8* was involved in leaf water loss and foliar ABA accumulation.

### Suppressed actin filaments activity causes increased abscisic acid accumulation

**Figure 7:**
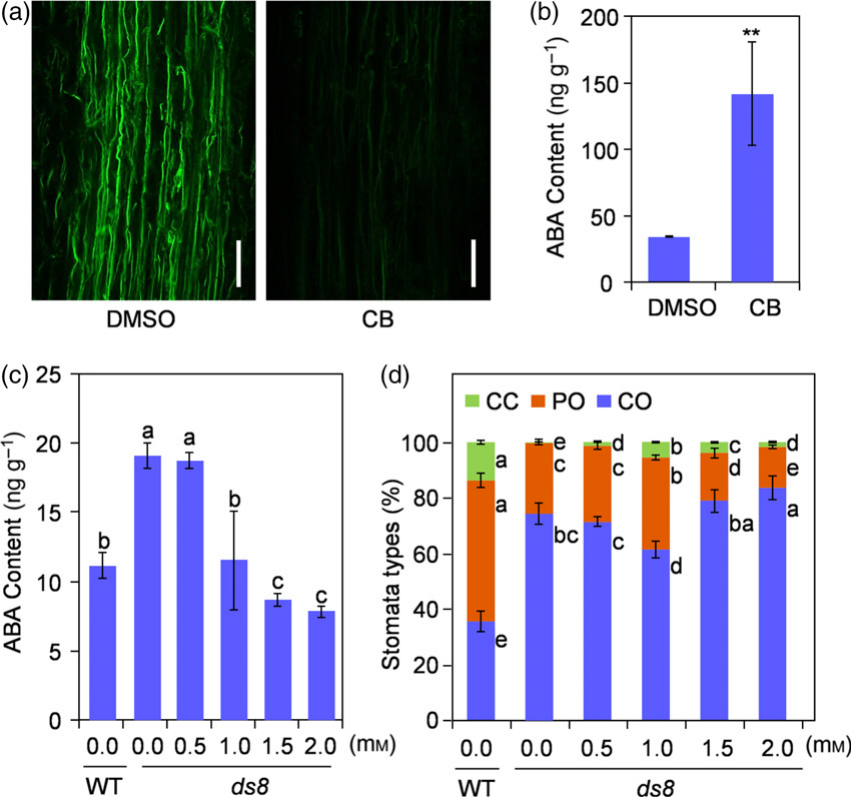
Suppressed actin filaments activity leads to increased abscisic acid (ABA) accumulation. (a) Actin filaments in the maturation zones of wild‐type (WT) root tips after 0.05% dimethylsulfoxide (DMSO; left) and 5 μm cytochalasin B (CB; dissolved in 0.05% DMSO) treatment (right) for 4 days. Scale bar: 20 μm. (b) Foliar ABA content after 0.05% DMSO and 5 μm CB treatment for 4 days. Data are the mean values. Data are represented as mean ± SD (*n *= 3). **P ≤ 0.01; Student's *t*‐test. (c) Foliar ABA content after treatment with different concentrations of sodium tungstate in an incubator. Data are represented as mean ± SD (*n *= 3). Different letters above columns indicate statistically significant differences (least significance difference test, *P *≤ 0.05). (d) Percentage of each type of stoma in WT and *ds8* after treatment with different concentrations of sodium tungstate. At least 100 stomata from five individual plants were analyzed for different treatments, respectively. Data are represented as mean ± SD. Different letters next to columns indicate statistically significant differences in each treatment of the same stoma type (least significance difference test, *P *≤ 0.05). CC, completely closed; PO, partially open; CO, completely open.

We treated 13‐day‐old wild‐type seedlings with 5 μm CB, an inhibitor of actin polymerization; 0.05% dimethylsulfoxide (DMSO) treatment was used for the control. Although 0.05% DMSO treatment led to disordered actin filaments to a certain extent, the number of actin filaments in CB‐treated root tips was clearly reduced compared with the control (Figure 7a). The ABA content of the seedling leaves was 33.97 ng g^−1^ on the 4th day after 0.05% DMSO treatment, whereas it was approximately 141.73 ng g^−1^ after CB treatment (Figure 7b). The foliar ABA content increased when the activity of actin filaments was suppressed.

On the other hand, *ds8* seedlings were treated with various concentrations of sodium tungstate, an ABA biosynthesis inhibitor (Figure 7c). The ABA content in *ds8* seedling leaves decreased with the increase of sodium tungstate concentration. Unexpectedly, the degree of stomatal closure in *ds8* leaves increased in response to decreased ABA content, reaching a maximum when the ABA content was similar to that of wild‐type (when the concentrations of sodium tungstate was 1.0 mm). With further decline of ABA content in response to a higher concentration of sodium tungstate (1.5 mm and 2.0 mm), the degree of stomatal closure in *ds8* leaves turned back to a decrease (Figure 7d).

## Discussion

In plants, SCAR/WAVE complex subunits were generally reported to affect cell morphogenesis by regulating actin filaments nucleation and branching. Their functions in drought stress were rarely mentioned (Brembu *et al*., 2004; Salah *et al*., 2004; Le *et al*., 2006; Panteris *et al*., 2009). In this study, we isolated and characterized the rice mutant *ds8*, which exhibited increased sensitivity to drought stress. *DS8* encoded a NAP1‐like protein, a component of the SCAR/WAVE complex. *DS8* is reported to encode an interacting protein of LPL2, a PIROGI/Specifically Rac1‐associated protein 1 (PIR/SRA1)‐like protein involved in pavement cell morphology. Its T‐DNA insertion mutant exhibits similar deficient pavement cell morphology to *lpl2* (Zhou *et al*., 2016). Here, we further cloned and identified *DS8* by functional complementation analysis and revealed its function in affecting drought sensitivity.

### ***DS8*** **is involved in actin filaments modeling**

In Arabidopsis, *GNARLED* encodes a NAP1 homolog that is reported to positively regulate the function of SCAR/WAVE‐Arp2/3 complex in the process of actin filaments nucleation and branching (Machesky *et al*., 1999; Deeks *et al*., 2004; Salah *et al*., 2004). In this study, DS8 shared high sequence similarity with GNARLED, according to the multiple sequence alignment (Figure S5). Like most SCAR/WAVE subunit mutants, *ds8* exhibited an abnormal actin cytoskeleton (Figure 2f–h). These results indicated that *DS8* encoded a NAP1 homolog that was involved in the nucleation of actin filaments by contributing to SCAR/WAVE‐Arp2/3 complex activity.

### ***DS8*** **is involved in cuticle development**

The successful transition of extant land plants from aquatic to terrestrial habitats has largely depended on the evolutionary acquisition of unique adaptations to the terrestrial environment, such as the distinctive epidermal components (Chen *et al*., 2017). Disorganized actin filaments in plants with SCAR/WAVE complex deficiency results in changes in epidermal trichome (in Arabidopsis)/CP (in rice) and pavement cell morphology (Basu *et al*., 2004, 2005; Deeks *et al*., 2004; Djakovic *et al*., 2006; Bai *et al*., 2015; Rao *et al*., 2015; Zhou *et al*., 2016). In our study, we also found epidermal changes in *ds8* plants. The shrunken bulliform cells (Figure 4b) explained the wrinkled bulliform cell band (Figure 4a) observed on the upper leaf epidermis of the *ds8*. The wrinkled bulliform cell band decreased the width of the S‐ST‐BC unit on mutant leaf surface to a certain extent (Figure 4c), which increased the number of S‐ST‐BC units per unit area and finally increased stomatal density (Figure 4d). So, the increased stomatal density in *ds8* due to altered bulliform cell morphology was a byproduct of abnormal epidermal cell morphogenesis.

Unlike other SCAR/WAVE subunit mutants, the cuticle of *ds8* leaves was impaired according to the result of toluidine blue staining (Figure 3a). *ds8* exhibited altered cuticle wax components (Figure 3d) and cell wall deposition (Figure S6) in leaves. The changes in proportion of cuticular wax components (Figure 3d), which could cause defects in epicuticular wax (Yu *et al*., 2008; Qin *et al*., 2011; Mao *et al*., 2012; Zhu and Xiong, 2013; Gan *et al*., 2016; Wang *et al*., 2017), accounted for the damaged cuticle layer in *ds8*. Some rice mutants with abnormal cuticles have thicker but less dense cuticular layers when compared with wild‐type plants (Mao *et al*., 2012; Gan *et al*., 2016), which could also be observed in our study (Figure 3b,c). All these results suggested that *DS8* not only functioned in epidermal cell morphogenesis like other SCAR/WAVE subunits, but also influenced the development of the cuticle and cell wall. Therefore, *DS8* was important for building complete leaf epidermis to protect plants against non‐stomatal water loss.

### ***DS8*** **is required for abscisic acid‐induced stomatal closure**

Mechanistic studies of stomatal movement have demonstrated that actin filaments dynamics functions downstream of ABA‐regulated stomatal movement, and the inhibition of actin filaments nucleation or distribution reduces stomatal closure in response to ABA or H_2_O_2_ stimulation (Pei *et al*., 2000; Zhang *et al*., 2001; Gudesblat *et al*., 2007; Hardham *et al*., 2007; MacRobbie and Kurup, 2007; Gao *et al*., 2009; Kim *et al*., 2010; Zhao *et al*., 2011; Jiang *et al*., 2012; Li *et al*., 2014; Munemasa *et al*., 2015; Cao *et al*., 2016). So, the presence of abnormal actin filaments in *ds8* explained why the mutant leaves exhibited a reduced degree of stomatal closure during normal growth (Figure 5b). The stomatal closure defect in *ds8* could not be recovered by exogenous ABA or H_2_O_2_ treatment (Figure 5c) because the actin filaments activity downstream of ABA‐induced stomatal closure was damaged. Besides, this defect unexpectedly recovered marginally in response to a decline in ABA content and subsequently decreased in response to a further decrease in ABA content (Figure 7c,d). This revealed that the ABA content in *ds8* could even enhance the severity of the stomatal closure deficiency when it exceeds a certain content. All these results suggested that *DS8* was indispensable for ABA‐induced stomatal closure. When *DS8* was invalid, the impaired actin filaments activity interfered with the ABA‐mediated stomatal closure.

Although the SCAR/WAVE complex and Arp2/3 complex were reported to work together to regulate actin filaments modeling (Pollard, 2007; Yanagisawa *et al*., 2015), there are still some doubts about their functions. In Arabidopsis, mutants of Arp2/3 complex subunits show reduced sensitivity to ABA‐regulated stomatal closure (Jiang *et al*., 2012; Li *et al*., 2014). However, the mutant of PIR, a subunit of the SCAR/WAVE complex, shows normal sensitivity to ABA‐regulated stomatal closure. This contradiction leaves two explanations. Firstly, the residual SCAR/WAVE complex activity in the PIR mutant is sufficient for the activation of Arp2/3 complex, so the activity of actin filaments is not affected. Secondly, the SCAR/WAVE complex is not involved in ABA‐mediated stomatal closure (Isner *et al*., 2017). In our study, the insensitivity of *ds8* to ABA treatment (Figure 5c) prompted us to support the former possibility. Different subunits of the SCAR/WAVE complex may affect the actin filaments activity at different degrees.

During ABA‐mediated stomatal closure, there is a feedback path. The depolymerization of actin filaments is thought to activate Ca^2+^ channels in the plasma membrane, which directly activates NADPH oxidases, leading to H_2_O_2_ production (Sagi and Fluhr, 2001; Zhang *et al*., 2007; Li *et al*., 2014). We suspected that ABA may be the first factor activated by actin filaments in this feedback path, for ABA could also activate Ca^2+^ channels (Munemasa *et al*., 2015) and the ABA content in *ds8* leaf was higher compared with that in wild‐type (Figure 5d). To investigate whether the increased ABA content in *ds8* leaves was due to restricted actin filaments activity, we treated *ds8* plants with CB. The ABA content of the CB treatment group was almost four times that of the control after the treatment (Figure 7b). These results indicated that the increase in ABA content in *ds8* was caused by suppressed actin filaments activity. Thus, we propose that the feedback path may be firstly activated by the increase in ABA levels induced by actin filaments dynamics. On the other hand, ABA was reported to promote leaf senescence (Takasaki *et al*., 2015; Zhao *et al*., 2016; Miao *et al*., 2018). A series of alterations detected in *ds8* leaves, such as elevated H_2_O_2_ content, higher rates of electrolyte leakage, reduced chlorophyll content, decreased photosynthetic rate, etc. indicated that leaf senescence was promoted in *ds8* due to the elevated ABA content (Figures 5e–i and S7).

In conclusion, *DS8* encoded a NAP1‐like protein, a component of SCAR/WAVE involved in actin filaments activity. The dysfunction of *DS8* resulted in abnormal development of the leaf epidermis, including impaired cuticles and increased stomatal density. Moreover, the impaired actin cytoskeleton in *ds8* led to defective ABA‐mediated stomatal closure. All of these defects in leaf traits rendered *ds8* suffering excessive water loss and increased sensitivity to drought. The impaired actin filaments in *ds8* leaves promoted the accumulation of endogenous ABA, leading to accelerated leaf senescence. In summary, *DS8* affects the sensitivity of rice plants to drought stress. Our findings reveal a genetic and molecular mechanism for the involvement of *DS8* in the response of rice to drought stress.

## Experimental procedures

### Plant materials and culture conditions

Plants were grown in a paddy field at the China National Rice Research Institute in Hangzhou under normal irrigation throughout the growth period (5–8 cm shallow water depth during early vegetative stage; saturated soil condition during heading stage and early filling stage). For ABA content measurement, leaves of wild‐type and mutant plants were harvested at the seedling stage, heading stage and 15 days after flowering. Flag leaves of wild‐type and mutant plants were collected during the heading stage and filling stage for specific analysis.

### Drought tolerance test and leaf water loss rate measurements

For the drought tolerance test in seedlings, germinated seeds were sown in containers of soil, cultured in an incubator (12 h light at 30°C and 12 h dark at 25°C, 70% humidity) and watered regularly. Watering was stopped for 7–10 days during the three‐leaf stage, followed by regular watering for 10 days. The test was repeated at least three times and only one picture was taken. The survival rate was scored after recovery. For the drought tolerance test in the field, irrigation was stopped 30 days after the seedlings were transplanted into large outdoor cement‐bound plots. Traits were examined 40 days after flowering. For leaf water loss rate analysis, six flag leaves were harvested from six individual plants during the heading stage and incubated in water. The excised leaves were cultured in an incubator at 28°C and 60% humidity after removing the excess water. The leaves were weighed every 10 min. The test was repeated three times, six leaves were used each time.

### Morphological observation of actin filaments

Alexa Fluor 488‐phalloidin staining of actin filaments was performed as described previously (Olyslaegers and Verbelen, 1998). To observe actin filaments, root tips were excised from seedlings and washed twice with PEM buffer (0.1 m PIPES, 10 mm EGTA, 0.3 m mannitol, 5 mm MgSO_4_, pH 6.9). The tissues were fixed in 4% paraformaldehyde in PEM buffer for 1 h, and washed twice with PEM buffer. The samples were stained with 0.66 mm Alexa Fluor 488‐phalloidin (Life Technologies, Eugene, OR, USA) in PEM buffer for 1 h and visualized under a confocal laser‐scanning microscope (LSM 700, Zeiss, Oberkochen, Germany).

### Observation of cuticular layer and quantification of wax and cell wall components

For TEM observations of the cuticular layer, the middle parts of flag leaves were harvested during the heading stage, fixed in precooled fixation buffer (0.1 m phosphate buffer containing 2.5% glutaraldehyde, pH 7.0) for at least 8 h, washed using phosphate buffer (0.1 m, pH 7.0) and dehydrated using a graded ethanol series. The tissue samples were then transferred to acetone for 20 min, embedded with resin, sectioned, stained with uranyl acetate and lead citrate, and visualized by TEM (H‐7650, Hitachi, Tokyo, Japan). For wax components quantification, flag leaves were harvested during the heading stage. Wax components were quantified by the Metabolomics Facility of the Institute of Genetics and Developmental Biology, Chinese Academy of Sciences. The test was repeated four times, and flag leaves collected from six plants were used each time. The content of cell wall components was analyzed according to Zhong and Läuchli (1993). The test was repeated three times, and flag leaves collected from six plants were used each time.

### Scanning electron microscopy and paraffin section analysis

For scanning electron microscopy (SEM) analysis of the upper leaf epidermis, the middle parts of flag leaves were harvested during the heading stage, fixed in pre‐cooled fixation buffer (0.1 m phosphate buffer containing 2.5% glutaraldehyde, pH 7.0) for at least 8 h, washed using phosphate buffer (0.1 m, pH 7.0), and dehydrated using a graded ethanol series. The tissue samples were then transferred to isoamyl acetate, dried, coated with gold and visualized by SEM (Hitachi TM‐1000). For paraffin section analysis, the middle parts of flag leaves were collected from wild‐type and mutant plants, and fixed in 50% FAA (0.9 m glacial acetic acid, 3.7% formaldehyde and 50% ethanol) overnight at 4°C. The paraffin section analysis was performed according to Ren *et al*. (2016).

### Analysis of stomatal aperture status and abscisic acid treatment

To analyse the stomatal aperture status of wild‐type and mutant leaves, the seedlings cultured under field conditions were used. The middle part of the upper leaf epidermis was covered with transparent nail polish and allowed to dry for about 5 min. The nail polish cast, which could provide an impression of the leaf surface, was removed from the leaf and attached to a microscope slide with transparent adhesive tape (Peel *et al*., 2017). The stomatal apertures status was observed under a 90i light microscope (Nikon, Tokyo, Japan) at 10 × 40 amplification. Stomatal types were defined and analyzed according to Matsuda *et al*. (2016).

For ABA and H_2_O_2_ treatment, germinated seeds were sown in reconstructive 96‐well plates and cultivated in distilled water for 3 days (12 h light at 30°C and 12 h dark at 25°C, 70% humidity). The seedlings were transferred to 1/2 Kimura B nutrient solution (Ueno *et al*., 2009) and cultivated for 10 days without changing the environmental conditions. Leaves were excised from 13‐day‐old seedlings and incubated in MES buffer (30 mm KCl, 0.1 mm CaCl_2_, 10 mm MES‐Tris, pH 6.1) for 90 min in the light (400 μmol m^−2 ^sec^−1^) to induce the maximum level of stomatal opening. Leaves with open stomata (used as the control) were transferred to MES buffer containing 30 μm ABA or 10^−4^ m H_2_O_2_ for 1 h (Li *et al*., 2014). The stomatal aperture status was analyzed according to the method mentioned in the previous paragraph.

### Measurement of abscisic acid, reactive oxygen species content and electrolyte leakage

To measure ABA content, fresh leaf samples were weighed and ground in liquid nitrogen. The powdered samples were combined with isopropanol‐hydrochloric acid extraction buffer for 30 min at 4°C. After the addition of methylene chloride, the suspension was mixed for 30 min at 4°C and centrifuged to isolate the organic phase. The organic phase was dried under liquid nitrogen and dissolved in methyl alcohol, which contained 0.1% formic acid. The final lysate was filtered and used for HPLC‐MS/MS. For the analysis of ROS content, leaf tissues were harvested during the filling stage. A rough estimate of superoxide anion and hyperoxide content was performed by NBT and DAB staining (Wu *et al*., 2016). The H_2_O_2_ content, CAT activity and electrolyte leakage were determined according to methods mentioned in previous studies (Moradi and Ismail, 2007; Morita *et al*., 2009).

### Measurement of net photosynthetic rate and pigment content

During filling stage, the photosynthetic rates of the flag leaves were measured using a Li‐6400 portable photosynthesis system (Li‐Cor, Lincoln, NE, USA) under the following conditions: 400 μmol mol^−1^ ambient CO_2_; 1200 μmol m^−2 ^sec^−1^ photosynthetic photon flux density and 500 μmol sec^−1^ air flow rate. The chlorophyll content was determined by spectrophotometry (Ye *et al*., 2016). The middle parts of flag leaves were collected from wild‐type and mutant plants cut into segments of about 1 cm each. The leaf samples were immersed in 80% acetone for 24 h (26°C in dark) and measured by DU800 ultraviolet spectrophotometer (Beckman, Fullerton, CA, USA).

### Sodium tungstate and cytochalasin B treatment

For sodium tungstate treatment, germinated seeds were sown in reconstructive 96‐well plates and placed in an incubator (12 h light at 30°C and 12 h dark at 25°C, 70% humidity), cultivated in distilled water for 3 days and in 1/2 Kimura B nutrient solution for 10 days. Then, the 13‐day‐old mutant seedlings were transferred to 1/2 Kimura B nutrient solution containing 0, 0.5, 1.0, 1.5 or 2.0 mm sodium tungstate for 7 days. Thirteen‐day‐old wild‐type seedlings were transferred to 1/2 Kimura B nutrient solution containing 0 mm sodium tungstate as a control. For CB treatment, 13‐day‐old wild‐type seedlings were transferred to 1/2 Kimura B nutrient solution containing 0.05% DMSO or 5 μm CB (dissolved in 0.05% DMSO). The ABA content and stomatal aperture status were analyzed according to the before‐mentioned methods in ‘Measurement of ABA, ROS content and electrolyte leakage’ and ‘Analysis of stomatal aperture status and ABA treatment’.

### Construct generation and transformation

For the complementation test, a 14.6‐kb genomic fragment containing the entire ORF of *LOC_Os08g43130* and its native promoter were amplified from NPB genomic DNA and introduced into binary vector pCAMBIA1300 to generate the *proDS8:DS8* plasmid. The plasmid was used to transform *ds8* calli by Agrobacterium‐mediated transformation. A GUS reporter construct containing the native *DS8* promoter was introduced into wild‐type rice plants to examine the expression pattern of *DS8*. To determine the subcellular localization of DS8, a *p35S:DS8‐eGFP* construct containing the full‐length *DS8* CDS without a stop codon was generated. Both the empty *p35S:eGFP* vector (as a control) and the *p35S:DS8‐eGFP* construct were introduced into wild‐type rice protoplasts and calli. A *DS8*‐antisense construct containing the reverse *DS8* CDS fragment under the control of the 35S promoter was generated and transformed into the wild‐type to produce *DS8* antisense transgenic lines. The primer sequences used are listed in Table S3.

### Accession numbers

Sequence data from this article can be found in the NCBI website (http:http://www.ncbi.nlm.nih.gov) under the following accession numbers: Os06g0130900 (*Histone H3*), Os08g0544500 (*DS8*).

## Conflict of interest

The authors declare no conflict of interests.

## Supporting information


**Figure S1.** Dysfunction of *DS8* increases the negative effects of a dry environment on rice production.

**Figure S2.** Phenotypes of various rice lines.

**Figure S3.** Expression pattern of *DS8* and subcellular localization of DS8.

**Figure S4.** *DS8* encodes a putative NAP1‐like protein.

**Figure S5.** Protein sequence alignment of DS8 and its homologs from several species.

**Figure S6.** Analysis of cell wall components and monosaccharide content.

**Figure S7.** *ds8* exhibits withered leaf tips and reduced chlorophyll content.

**Table S1.** Phenotypic data of wild‐type and *ds8* under normal and dry environmental conditions.
**Table S2.** Reciprocal crosses between *ds8* and wild‐type *indica* cultivars.
**Table S3.** Primers used in this study.
**Table S4.** Water loss rates of excised leaves harvested from transgenic complementation lines at the heading stage.
**Table S5.** Water loss rates of excised leaves harvested from WT, *ds8* and antisense transgenic plants at the heading stage.
