# Supplementary figures and images for: A Nck‐associated protein 1‐like protein affects drought sensitivity by its involvement in leaf epidermal development and stomatal closure in rice
Source: Plant J. 2019 Mar 18;98(5):884–97. doi: 10.1111/tpj.14288 (PMC6849750; doi:10.1111/tpj.14288)

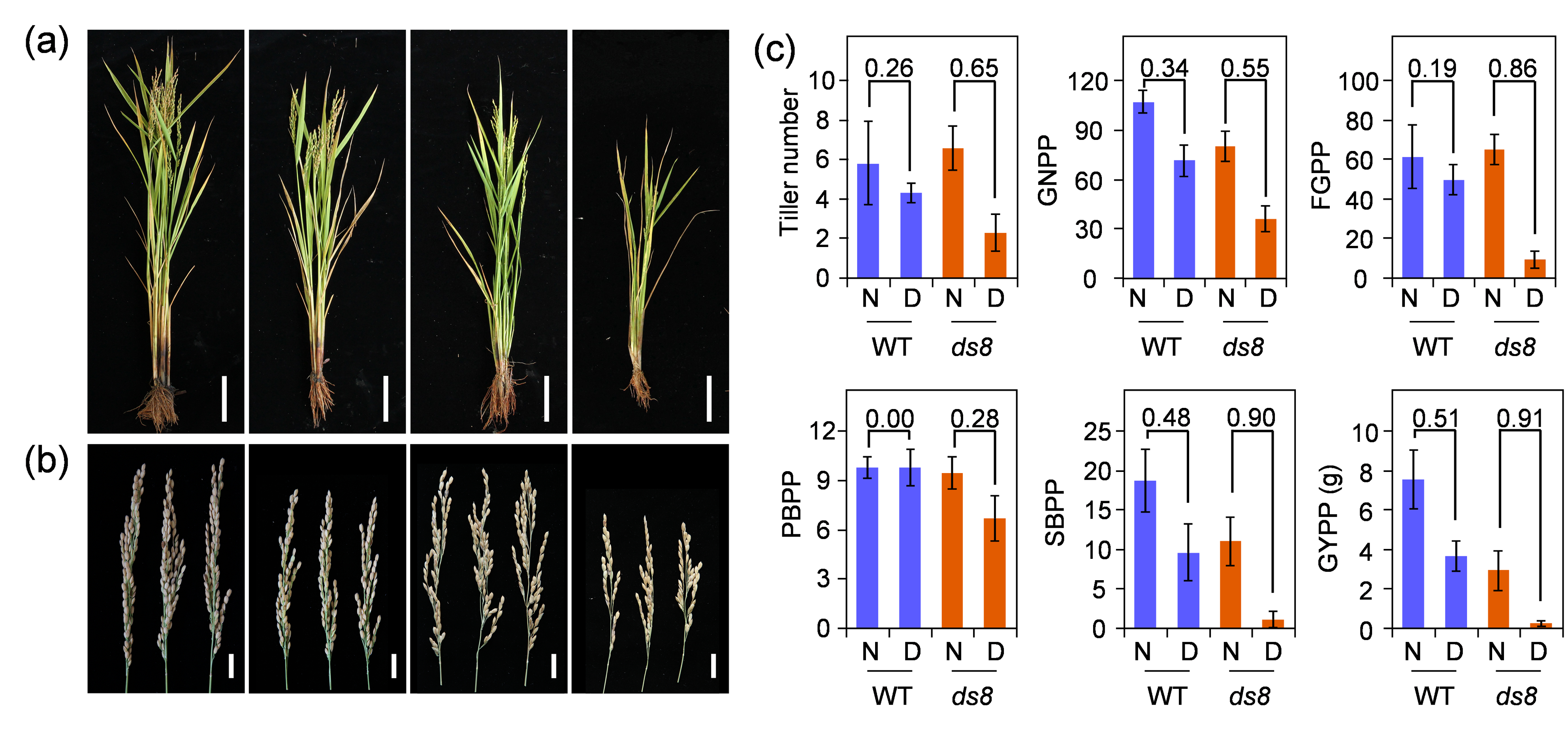

Supplement: Supplementary file 1 — Figure S1. Dysfunction of DS8 increases the negative effects of a dry environment on rice production. [file TPJ-98-884-s001.tif]

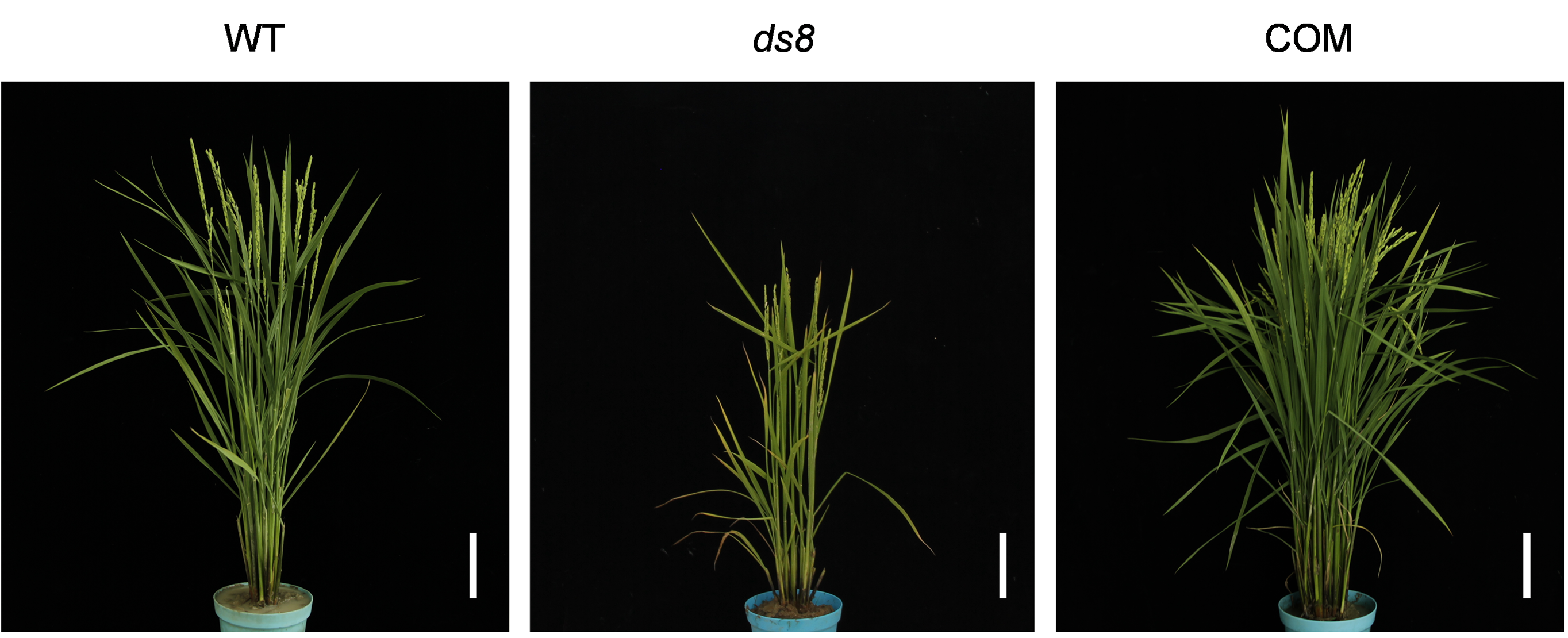

Supplement: Supplementary file 2 — Figure S2. Phenotypes of various rice lines. [file TPJ-98-884-s002.tif]

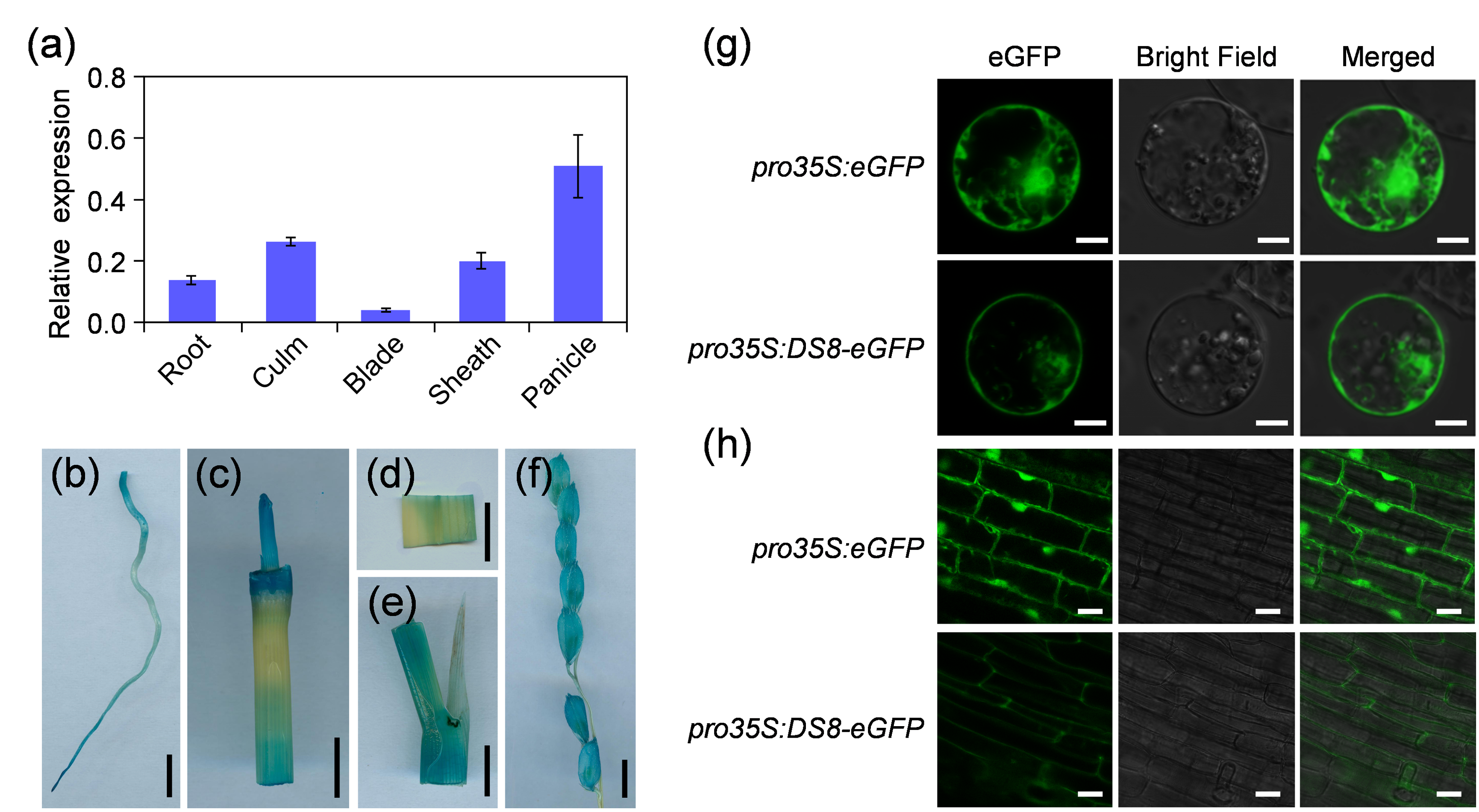

Supplement: Supplementary file 3 — Figure S3. Expression pattern of DS8 and subcellular localization of DS8. [file TPJ-98-884-s003.tif]

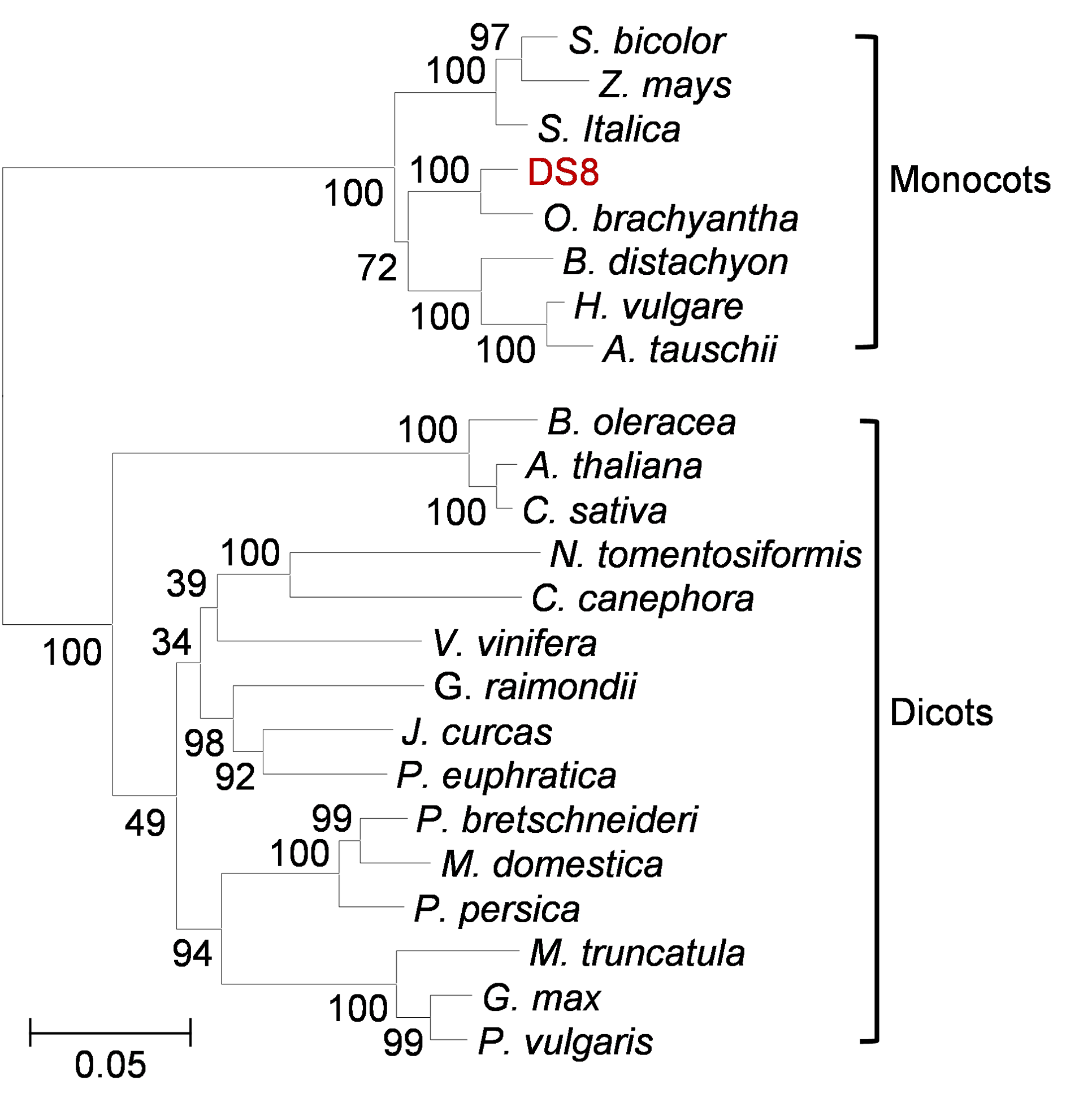

Supplement: Supplementary file 4 — Figure S4. DS8 encodes a putative NAP1‐like protein. [file TPJ-98-884-s004.tif]

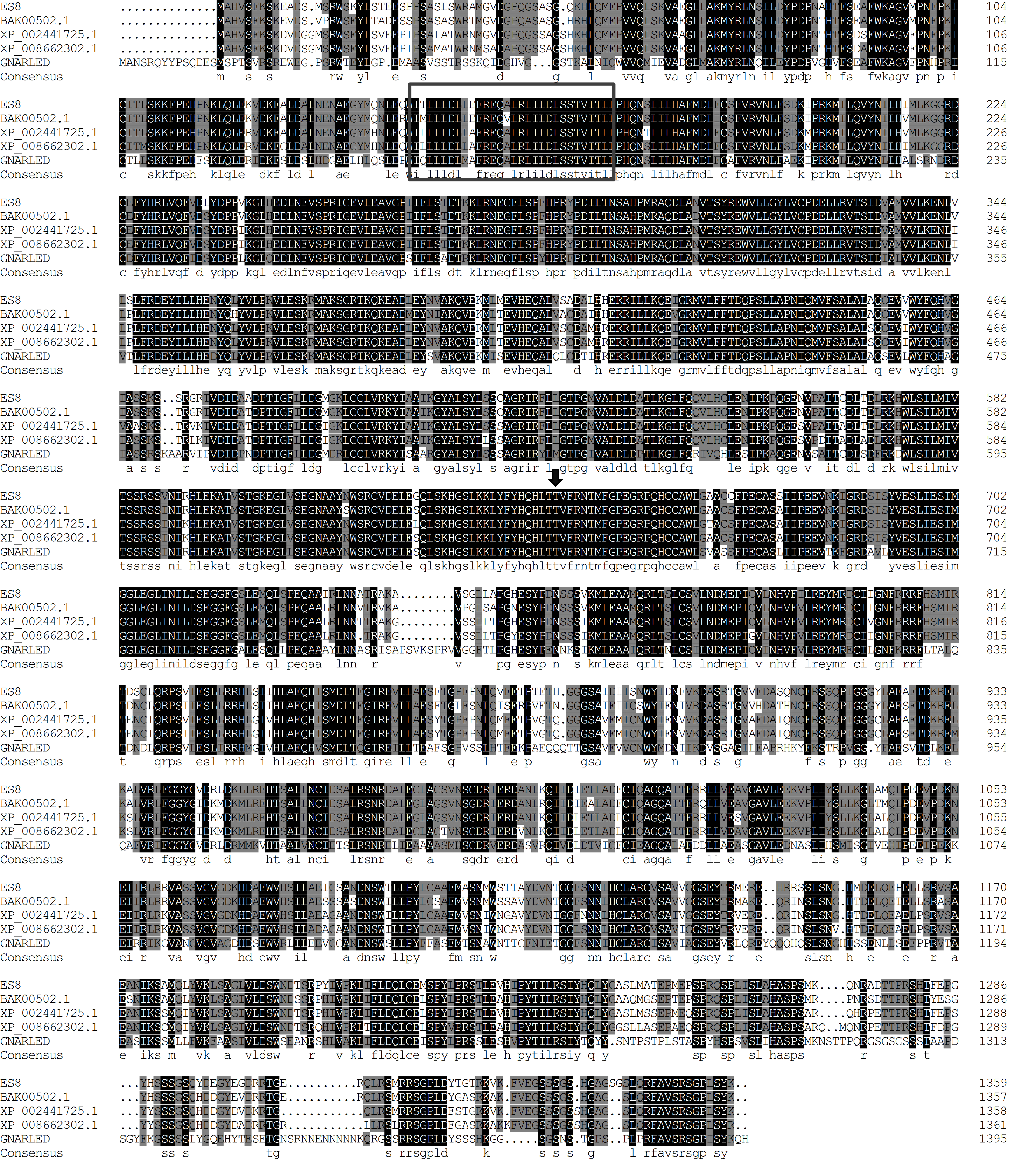

Supplement: Supplementary file 5 — Figure S5. Protein sequence alignment of DS8 and its homologs from several species. [file TPJ-98-884-s005.tif]

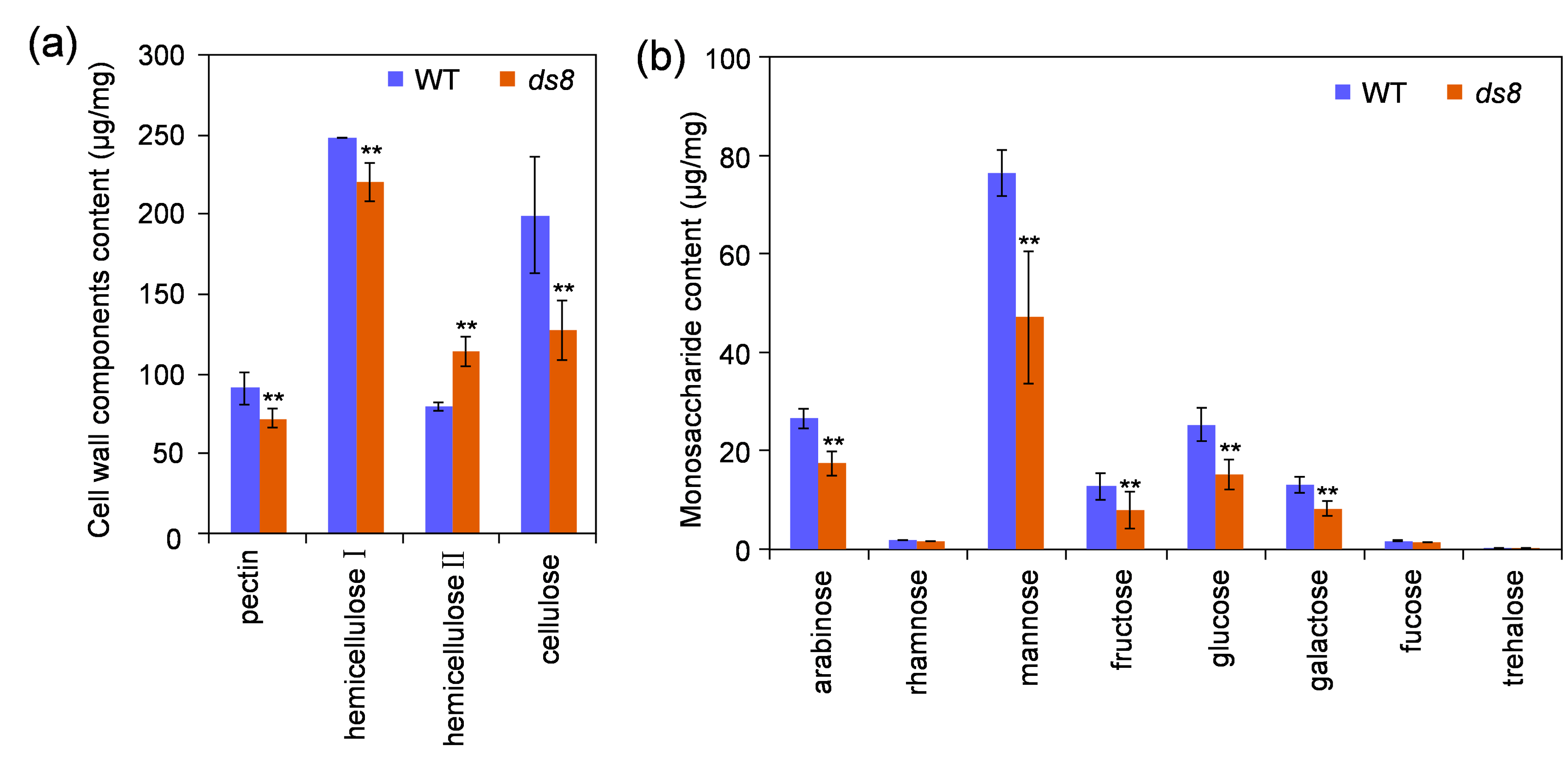

Supplement: Supplementary file 6 — Figure S6. Analysis of cell wall components and monosaccharide content. [file TPJ-98-884-s006.tif]

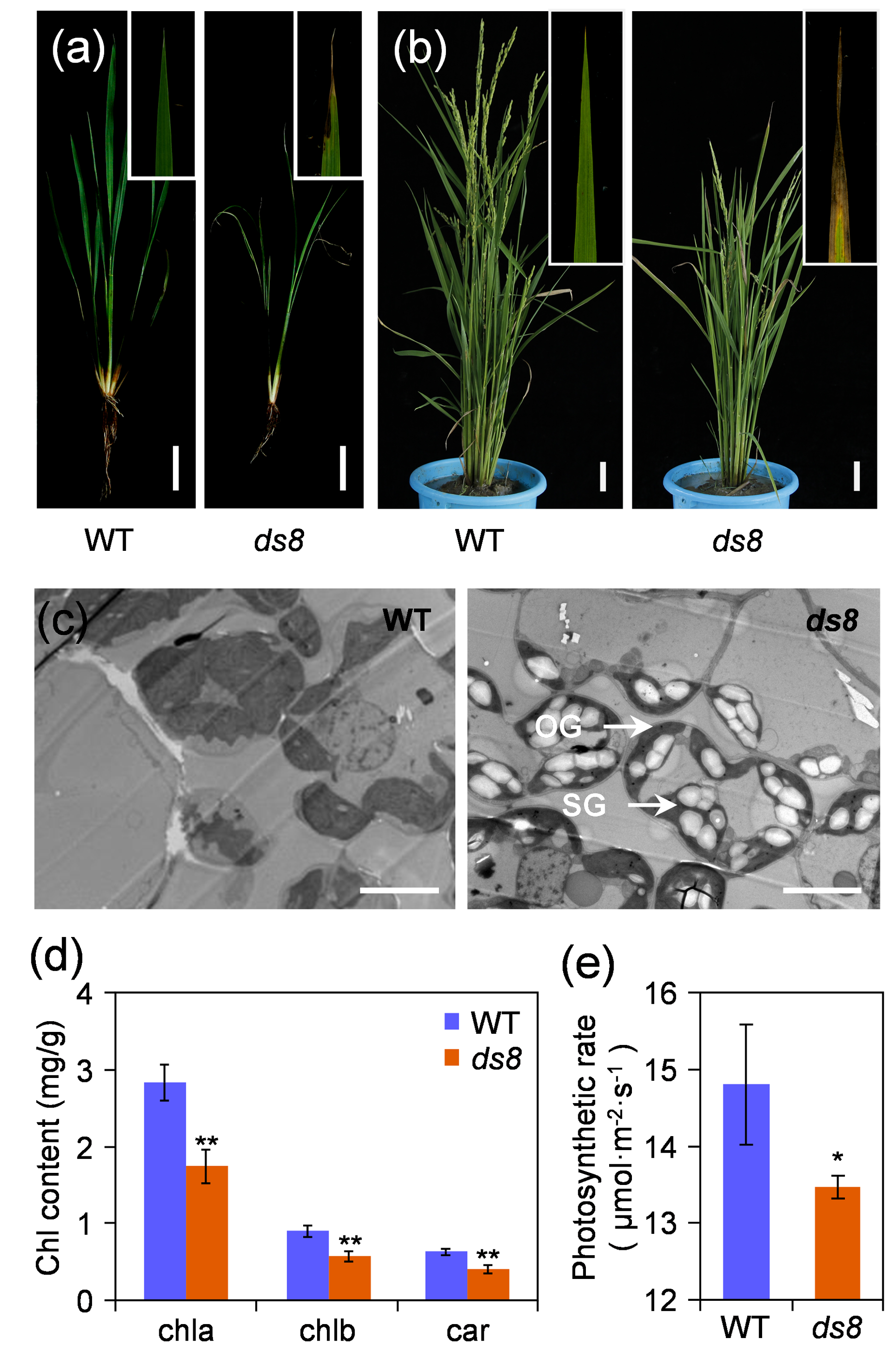

Supplement: Supplementary file 7 — Figure S7. ds8 exhibits withered leaf tips and reduced chlorophyll content. [file TPJ-98-884-s007.tif]
