# Supplementary material for: A Nck‐associated protein 1‐like protein affects drought sensitivity by its involvement in leaf epidermal development and stomatal closure in rice
Source: Plant J. 2019 Mar 18;98(5):884–97. doi: 10.1111/tpj.14288 (PMC6849750; doi:10.1111/tpj.14288)
Supplement: Supplementary file 8 — Table S1. Phenotypic data of wild‐type and ds8 under normal and dry environmental conditions. Table S2. Reciprocal crosses between ds8 and wild‐type indica cultivars. Table S3. Primers used in this study. Table S4. Water loss rates of excised leaves harvested from transgenic complementation lines at the heading stage. Table S5. Water loss rates of excised leaves harvested from WT, ds8 and antisense transgenic plants at the heading stage. [file TPJ-98-884-s008.doc]

**Table S1. Phenotypic data of wild type and *dt8* under normal and dry environmental conditions.**

| Trait |  | WT | | | | |  | *dt8* | | | | |
| --- | --- | --- | --- | --- | --- | --- | --- | --- | --- | --- | --- | --- |
|  |  | Normal |  | Drought |  | Decline Rate |  | Normal |  | Drought |  | Decline Rate |
| Tiller number |  | 5.8±2.10 |  | 4.3±0.48 |  | 25.86% |  | 6.6±1.13 |  | 2.3±0.95 |  | 65.00% |
| GNPP |  | 107.2±6.91 |  | 71.3±9.36 |  | 33.51% |  | 80.0±9.21 |  | 36.2±7.91 |  | 54.77% |
| FGPP |  | 61.4±15.99 |  | 49.8±7.45 |  | 18.99% |  | 64.9±7.56 |  | 9.4±4.41 |  | 85.55% |
| PBPP |  | 9.8±0.67 |  | 9.8±1.09 |  | 0.00% |  | 9.4±0.98 |  | 6.7±1.38 |  | 28.79% |
| SBPP |  | 18.7±3.92 |  | 9.6±3.66 |  | 48.53% |  | 11.0±3.11 |  | 1.1±1.07 |  | 89.61% |
| GYPP (g) |  | 7.5±1.46 |  | 3.7±0.77 |  | 51.41% |  | 2.9±1.01 |  | 0.3±0.15 |  | 91.17% |

**Table S2. Reciprocal crosses between *dt8* and wild-type *indica* cultivars.**

| Cross combination | F_1_ | |  | F_2_ | | | | |
| --- | --- | --- | --- | --- | --- | --- | --- | --- |
|  | Wild-type | Mutant |  | Wild-type | Mutant | Total | P-value | χ2（3:1） |
| *dt8*/9311 | 18 | 0 |  | 161 | 49 | 210 | 0.577 | 0.3111 |
| 9311/*dt8* | 15 | 0 |  | 221 | 69 | 290 | 0.635 | 0.2253 |
| *dt8*/ZF802 | 19 | 0 |  | 518 | 170 | 688 | 0.8602 | 0.031 |
| ZF802/*dt8* | 15 | 0 |  | 311 | 92 | 403 | 0.3141 | 1.0132 |
| *dt8*/CJ06 | 10 | 0 |  | 251 | 76 | 327 | 0.4627 | 0.5392 |
| CJ06/*dt8* | 11 | 0 |  | 181 | 50 | 231 | 0.239 | 1.3867 |

**Table S3. Primers used in this study.**

| Primer name | Forward primer | Reverse primer | Use |
| --- | --- | --- | --- |
| RM3120 | ATCGATGGAAGCTCTTTGCC | GGATGTACAAGAGCTTAGGAGC | Fine mapping |
| RM1345 | ACCACCACGCCATTAGAGAC | TGAGCATCCCGTGCTGTC | Fine mapping |
| M1 | ACCCATCATGCATCAGTCAC | GATCAGATCAGTACGCTTTTGC | Fine mapping |
| M2 | CCAGACCACTGGATGCACT | CTTGTCACCGCCGTAAAGG | Fine mapping |
| nm1 | ATCATGCATAAATGGGCATC | TCAATGAAAAACCCGCATGT | Fine mapping |
| nm2 | TATTGGTTTCTTGGCGACGC | ACAGATCGTCGAGTGGTACA | Fine mapping |
| nm3 | AGGAAGTAAGTTCCCTCGGAAT | GCAGCATAAAGCACCTGAAG | Fine mapping |
| nm4 | ATTCTGAATCCGGCCAAAG | AGTCGGCAGCTACCTTGAAT | Fine mapping |
| nm5 | CATGATTTTAACCAGGTCAATT | CGTTTCCCAGTTCAGTTTCG | Fine mapping |
| E1 | CATATGGGCTCCATCGATCT | GCATGCTCGAAGAAGACAAA | Fine mapping |
| E2 | GGTCCGGCTCCTAGGTT | GGCTGTGACGATCGATGAAT | Fine mapping |
| *DT8*-*COM* | GACCTGCAGGCATGCAAGCTACCTGTAAACTGCGAGACGA | CGACGGCCAGTGCCAAGCTTCCTCTCCATCAAGCACCTCA | Complementation analysis |
| *DT8*-*GUS* | GGTACCGTGTCATACTGTGGCTATGTTTAGTTT | CCATGGGTTTTGAGTACAGAACAACTGCATGG | Expression pattern analysis |
| *DT8*-*GFP* | GGATCCATGGCCCATGTTTCGTTCAAATCC | AAGGATCCTTTGTAGGATAGGGGGCCAGATCTT | Subcellular localization analysis |
| *DT8*-*Anti* | GCAGGTCGACTCTAGATGGCCCATGTTTCGTTCAAAT | CCGGGGATCCTCTAGTTATTTGTAGGATAGGGGGCCA | Antisense transgenic lines analysis |
| P1 | ATCCAGATCCAAATGCGCAC | CCCCTTGACTGGTGGATCAT | CDS differentiation test |
| P2 | TGTGCTGGAAGAATACGGTT | TGTGTTTCTGAAGACCGTTGT | CDS differentiation test |
| *Lsi1* | CTCCTCCCAGAAGCTCTCCT | TCTCGTCGTCCTATCACACT | Real-time PCR |
| *DT8* | CACAACGCCCAGGTCTCATACT | TGCTTCGGAGTTGCCTCTCTC | Real-time PCR |
| *Histone H3* | GGTCAACTTGTTGATTCCCCTCT | AACCGCAAAATCCAAAGAACG | Real-time PCR |

**Table S4.** Water loss rates of excised leaves harvested from transgenic complementation lines at the heading stage.

| Time (min) | NPB | ds8 | COM-1 | COM-2 |
| --- | --- | --- | --- | --- |
| 10 | 11.10 b^*^ | 14.94 a | 11.68 b | 10.93 b |
| 20 | 19.69 b | 23.38 a | 17.17 b | 17.01 b |
| 30 | 24.88 b | 29.36 a | 22.22 b | 22.49 b |
| 40 | 29.59 b | 38.29 a | 27.69 b | 27.97 b |
| 50 | 34.64b | 46.62 a | 34.24 b | 34.68 b |
| 60 | 40.46 b | 56.81 a | 41.54 b | 41.57 b |
| 70 | 46.14 b | 66.24 a | 47.49 b | 47.67 b |
| 80 | 50.66 b | 73.24 a | 53.60 b | 52.51 b |
| 90 | 55.24 b | 81.56 a | 57.89 b | 58.30 b |
| 100 | 59.79 b | 88.32 a | 62.90 b | 63.45 b |
| 110 | 65.87 b | 95.07 a | 69.50 b | 70.16 b |
| 120 | 73.73 b | 98.49 a | 77.42 b | 78.30 b |
| 130 | 77.94 b | 99.23 a | 81.70 b | 82.68 b |
| 140 | 81.42 b | 100.00a | 84.86 b | 86.11 b |
| 150 | 86.84 b | 100.00a | 89.98 b | 90.80 b |

^*^ Within a row, means followed by different letter are significantly different at 0.05 probability level according to LSD test.

**Table S5.** Water loss rates of excised leaves harvested from WT, *ds8*, and antisense transgenic plants at the heading stage.

| Time (min) | NPB | ds8 | Anti-1 | Anti-2 |
| --- | --- | --- | --- | --- |
| 10 | 11.44 b^*^ | 12.51 ba | 11.84 b | 17.99 a |
| 20 | 20.27 a | 20.33 a | 19.81 a | 23.53 a |
| 30 | 24.64 b | 26.68 a | 25.45 ba | 29.40 ba |
| 40 | 29.91 b | 34.65 a | 31.37 b | 33.32 ba |
| 50 | 34.13 b | 43.28 a | 38.40 b | 38.57 b |
| 60 | 40.02 c | 52.94 a | 45.92 b | 44.64 bc |
| 70 | 45.31 c | 61.58 a | 53.30 b | 50.96 bc |
| 80 | 49.93 c | 69.19 a | 58.23 b | 56.17 b |
| 90 | 54.38 c | 77.66 a | 64.32 b | 62.28 b |
| 100 | 59.02 c | 84.85 a | 70.24 b | 67.81 b |
| 110 | 64.49 c | 92.05 a | 76.78 b | 73.77 b |
| 120 | 72.96 c | 96.67 a | 84.89 b | 82.92 b |
| 130 | 77.36 c | 98.46 a | 89.03 b | 86.53 b |
| 140 | 80.73 c | 99.48 a | 91.88 b | 90.57 b |
| 150 | 86.00 c | 100.00 a | 95.30 b | 94.50 b |

^*^ Within a row, means followed by different letter are significantly different at 0.05 probability level according to LSD test.
