# Supplementary material for: A Nck‐associated protein 1‐like protein affects drought sensitivity by its involvement in leaf epidermal development and stomatal closure in rice
Source: Plant J. 2019 Mar 18;98(5):884–97. doi: 10.1111/tpj.14288 (PMC6849750; doi:10.1111/tpj.14288)
Supplement: Supplementary file 9 [file TPJ-98-884-s009.docx]

**Legends for Supporting Figures**

**Figure S1.** Dysfunction of *DS8* increases the negative effects of a dry environment on rice production.

(a) Plants grown under drought-stress conditions in the field. From left to right: WT (normal conditions), WT (drought conditions), *ds8* (normal conditions) and *ds8* (drought conditions). Bar=10 cm.

(b) Panicles of plants grown under drought-stress conditions in the field. From left to right: WT (normal conditions), WT (drought conditions), ds8 (normal conditions) and *ds8* (drought conditions). Bar=2 cm.

(c) WT and *ds8* after normal and drought-stress treatment in the field. N, normal conditions; D, drought conditions; GNPP, grain number per panicle; FGPP, filled grains per panicle; PBPP, primary branch per panicle; SBPP, secondary branch per panicle; GYPP, grain yield per plant. The decline rates are labled. Data are represented as mean ± SD (*n*=6).

**Figure S2.** Phenotypes of various rice lines. Phenotypes of WT (left), *ds8* (middle), and transgenic complementation plant (right) during the heading stage. Bar=10 cm.

**Figure S3.** Expression pattern of *DS8* and subcellular localization of DS8.

(a) Quantitative RT-PCR analysis of *DS8* transcript levels in various tissues at the booting stage. Data are the mean values. Error bars indicate SD of three biological repeats.

(b-f) GUS staining of root (b), culm (c), blade (d), sheath (e), and panicle tissue (f). Bar=1 cm.

(g) Transient expression of DS8-eGFP fusion protein in rice protoplasts. Bar=5 μm.

(h) Subcellular localization of DS8-eGFP fusion protein in the roots of *pro35S:DS8-eGFP* transgenic plants. Bar=10 μm.

**Figure S4.** *DS8* encodes a putative NAP1-like protein.

(a) Phylogenetic analysis of DS8 homologs. The following protein sequences were used to construct the phylogenetic tree: *Brassica oleracea* XP 013636313.1; *Arabidopsis thaliana* GNARLED; *Camelina sativa* XP 010516680.1; *Nicotiana tomentosiformis* XP 009593115.1; *Coffea canephora* CDP11252.1; *Vitis vinifera* XP 002276461.1; *Gossypium raimondii* XP 012467099.1; *Jatropha curcas* XP 012065958.1; *Populus euphratica* XP 011029922.1; *Pyrus x bretschneideri* XP 009353971.1; *Malus domestica* XP 008371895.1; *Prunus persica* XP 007213730.1; *Medicago truncatula* XP 003607894.2; *Glycine max* XP 003556250.1; *Phaseolus vulgaris* XP 007157772.1; *Sorghum bicolor* XP 002441725.1; *Zea mays* XP 008662302.1; *Setaria italica* XP 012702495.1; DS8; *Oryza brachyantha* XP 006659651.1; *Brachypodium distachyon* XP 003574876.1; *Hordeum vulgare* BAK00502.1; *Aegilops tauschii* EMT06681.1.

**Figure S5.** Protein sequence alignment of DS8 and its homologs from several species. Protein sequence alignment was performed among DS8 and its homologs from *Hordeum vulgare*, *Sorghum bicolor*, *Zea mays* and *Arabidopsis thaliana* using DNAMAN. White letters on a black background refer to amino acids conserved across all these homologs. Amino acids framed in box show the missing part of the mutant protein. Arrow indicates the point where the frameshift starts.

**Figure S6.** Analysis of cell wall components and monosaccharide content.

(a) Comparison of cell wall component content between WT and *ds8*. Data are represented as mean ± SD (*n*=3). ***P* ≤ 0.01; Student’s *t*-test.

(b) Monosaccharide content in WT and *ds8* leaves (*n*=3). Data are represented as mean ± SD (*n*=3). ***P* ≤ 0.01; Student’s *t*-test.

**Figure S7.** *ds8* exhibits withered leaf tips and reduced chlorophyll content.

(a) Phenotypes of WT (left) and *ds8* (right) plants during the seedling stage. Bar=5 cm.

(b) Phenotypes of WT (left) and *ds8* (right) plants during the filling stage. Bar=5 cm.

(c) Transmission electron microscopy analysis of mesophyll cells in WT (left) and *ds8* (right) leaves during the filling stage. Bar=5 μm. OG, osmiophilic globule; SG, starch grain. Bar=5 μm.

(d) Chlorophyll content in WT and *ds8* during the filling stage. Data are represented as mean ± SD (*n*=4). ***P* ≤ 0.01; Student’s *t*-test.

(e) Comparison of photosynthetic rate between WT and *ds8* at the filling stage. Data are represented as mean ± SD (*n*=4). **P* ≤ 0.01; Student’s *t*-test.
